# The Evils of Substitution

**Published:** 1893-12

**Authors:** Cyrus Edson

**Affiliations:** Commissioner of Health of New York City and State; President of the Board of Pharmacy of the City and County of New York


### THE EVILS OF SUBSTITUTION.

By CYRUS EDSON, M. D.,

Commissioner of Health of New York City and State; President of the Board of Pharmacy of the City and County of New York.

The term "substitution," in its commercial sense, is the perpetration of a fraud by the seller upon the buyer, the former selling the latter something different from the article demanded, under the same name. This fraud is really but another phase of commercial adulteration, and in the practice of pharmacy its evils are as insidious and harmful as those of any crime committed by man. These evils are both direct and remote in their effects. They injure, first, the patient; second, the physician; third, the manufacturer. From the standpoint of the patient, the evil affects him directly and indirectly. The dishonest pharmacist has, of course, palmed off on his unsuspecting customer a cheaper preparation than that ordered by the prescriber, because the motive for the crime is, in ninety-nine cases out of a hundred, a mercenary one. The result to the patient from the inhibition of the substituted article may be one of the following: first, no therapeutic action; second, therapeutic action of less potency; third, therapeutic action of greater potency; fourth, therapeutic action of different character than aimed at by the prescriber. It needs no argument to prove that any of these four results would, under certain conditions, be likely to be disastrous to the patient.

The pharmacist is the responsible and trusted dispenser of the physician's order, and when he acts differently than ordered by the doctor, he snips at the threads of fate, possibly without the slightest idea of what will result from the snipping. Then, he is no better than a man who fires a bullet among a crowd of people. The result in either case may be manslaughter. Let us take a less extreme view of the crime from the patient's standpoint. The latter fails to get benefit from his medicine, and, as a result, loses time and money. He was cheated when he bought the preparation. Now, indirectly, he has lost the fee he paid the physician, and, last but not least, he has lost confidence in his doctor.

From the standpoint of the physician, the evils of substitution have a wider range in their effect than on the individual patient. Medicine has been said to be an inexact science. The reason of this is because it is very difficult to ascribe a given effect to a certain cause. In other words, so many causes operate to produce a given effect in the human economy that it is difficult to ascertain and fix upon a definite cause. Modern therapeutics is the outcome of the physician's observations and experience of the effect of drugs upon the human system. It is a science to which every physician contributes his mite or his much, according to his ability and his opportunity.

The pharmacist who substitutes leads physicians astray. By presenting false premises to the latter, the former causes him to make erroneous deductions. The entire medical profession may thus feel the result of a single instance of substitution, and numerous other invalids suffer on account of the errors following faulty experience in the case of the physician treating a single patient who is the victim of the fraud in question.

I have already spoken of the loss of confidence in his physician on the part of the victimized patient. This has not only a direct effect upon the invalid, because confidence in his doctor's efforts are, to a great extent, essential to the latter's success in the treatment of the case, but it may also cause the dismissal of the physician, and his loss of what, perhaps, would have been a lucrative practice. In this country, physicians have the reputation of being practical. They are the best practitioners in the world.

In other countries, medical men are deeper students and better theorists, but here we pride ourselves on the results we obtain in curing disease. The reason for this is because we strive less for honor and glory than we do for the almighty dollar. We must give our patients the worth of their money, and we know we will not be tolerated unless we do. Our patients are quick to discover mistakes, and they are laid at the door of the physician rather than at that of the pharmacist. If this was not the case, the subject of substitution would not be worth consideration, for it would be a rarely committed crime.

The question of injury to the manufacturer is a very important phase of the matter, for, rather singularly, the remedy for the great evil must spring mainly from this source. This is not so strange after all when we come to think of it, for here we find the effects of the evils of substitution so direct and so distinctly felt that interest is natural. Nothing causes men more concern than pecuniary loss. Cause and effect are here so closely associated that a hue and cry at once follows. The manufacturer invests large sums in producing a reliable preparation; he spends more in bringing it before the medical profession. The latter find it worthy of use and patronize it until the weeds of substitution check its growth. The way these weeds act, after what I have said, is obvious. For example, some pharmacist substitutes an inferior mixture or drug in the preparation of the physician's prescription; the effect of the medicine on his patient is *nil.* The disappointed doctor heralds the fact to his brethern. Such news travels faster than any favorable comments, and undoes in a short time that which the manufacturer has taken months, or perhaps years, to accomplish. Great injury is in consequence done to a deserving business.

Then again, the evil is a widespread one, and the same substitution in a good preparation is very large, and directly affects its sale. I know of no other crime that tends so much to destroy one's faith in man's goodness as substitution. For the sake of insignificant profit, the dishonest pharmacist deliberately cheats and perhaps destroys his fellow-man. I can only account for the practice by assuming that the perpetrator in some way persuades himself that he is doing no harm, that he is selling something "just as good," that he holds the judgment and knowledge of the physician in small repute, and that he feels perfectly competent to act in the premises. It is a curious psychological fact, that it is the easiest thing in the world for a man engaged in a nefarious trade to persuade himself that he is doing no harm so long as he is making money by his acts.

To correct the practice of substitution does not seem to me a difficult matter. A few years ago, the adulteration of food products was a very serious fraud. Confectionery, for example, was greatly adulterated at that time. The exposure of the practice by the Health Department of New York City so injured the confectionery business, that the reputable manufacturers banded together in an Anti-Adulteration League. Not only did the Health Department cause the formation of the league in the way I have described, but the unfair competition, engendered by adulteration, also had its effect in forcing honest manufacturers to protect themselves. This league made it its business to run down and punish all persons who adulterated their wares. The result was that in a short time adulteration ceased, and today it is impossible to find any adulterated candy offered for sale. Another instance of manufacturers banding together for mutual protection is offered by the Jewelers' Protective Association. This body pursues like an avenging Nemesis any one who robs or cheats its members. Let the manufacturers of pharmaceutical preparations who suffer from the evils of substitution, form a like union, and charge its agents with the duty of bringing to justice the perpetrators of the fraud of substitution. The Penal Code and the Pharmacy Act both afford excellent laws for the punishment of these criminals. The Board of Pharmacy is not sufficiently equipped to enforce the provisions of the law to this end, and the Health Department is too busily engaged fighting disease to cope with the evil. The formation of such a union as I have indicated, however, and the punishment of a few offenders would soon stop the practice. The mere publication of a few instances of fraud, giving the names and addresses of the dishonest pharmacists, would go far towards suppressing substitution, for the public is quick to discover and shun the druggist who is considered unreliable and unscrupulous.—*The Doctor of Hygiene.*

